# Accelerating ESD-induced gastric ulcer healing using a pH-responsive polyurethane/small intestinal submucosa hydrogel delivered by endoscopic catheter

**DOI:** 10.1093/rb/rbaa056

**Published:** 2021-01-04

**Authors:** Long-Mei Zhao, Mei Gong, Rui Wang, Qi-Juan Yuan, Yi Zhang, Jin-Kui Pi, Xiu-He Lv, Yan Xie, Hui-Qi Xie

**Affiliations:** Laboratory of Stem Cell and Tissue Engineering, Orthopedic Research Institute, State Key Laboratory of Biotherapy and Cancer Center, West China Hospital, Sichuan University and Collaborative Innovation Center of Biotherapy, Keyuan Road 4, Gaopeng Street, Chengdu, Sichuan 610041, China; Laboratory of Stem Cell and Tissue Engineering, Orthopedic Research Institute, State Key Laboratory of Biotherapy and Cancer Center, West China Hospital, Sichuan University and Collaborative Innovation Center of Biotherapy, Keyuan Road 4, Gaopeng Street, Chengdu, Sichuan 610041, China; Laboratory of Stem Cell and Tissue Engineering, Orthopedic Research Institute, State Key Laboratory of Biotherapy and Cancer Center, West China Hospital, Sichuan University and Collaborative Innovation Center of Biotherapy, Keyuan Road 4, Gaopeng Street, Chengdu, Sichuan 610041, China; Laboratory of Stem Cell and Tissue Engineering, Orthopedic Research Institute, State Key Laboratory of Biotherapy and Cancer Center, West China Hospital, Sichuan University and Collaborative Innovation Center of Biotherapy, Keyuan Road 4, Gaopeng Street, Chengdu, Sichuan 610041, China; Research Core Facility, West China Hospital, Sichuan University, Keyuan Road 4, Gaopeng Street, Chengdu, Sichuan 610041, China; Research Core Facility, West China Hospital, Sichuan University, Keyuan Road 4, Gaopeng Street, Chengdu, Sichuan 610041, China; Department of Gastroenterology, West China Hospital, Sichuan University, No. 37 Guo Xue Xiang, Chengdu, Sichuan 610041, China; Department of Gastroenterology, West China Hospital, Sichuan University, No. 37 Guo Xue Xiang, Chengdu, Sichuan 610041, China; Laboratory of Stem Cell and Tissue Engineering, Orthopedic Research Institute, State Key Laboratory of Biotherapy and Cancer Center, West China Hospital, Sichuan University and Collaborative Innovation Center of Biotherapy, Keyuan Road 4, Gaopeng Street, Chengdu, Sichuan 610041, China

**Keywords:** ESD-induced ulcer, small intestinal submucosa, polyurethane, bio-adhesion, pH-sensitive

## Abstract

Endoscopic submucosal dissection (ESD) is the standard treatment for early-stage gastric cancer, but the large post-operative ulcers caused by ESD often lead to serious side effects. Post-ESD mucosal repair materials provide a new option for the treatment of post-ESD ulcers. In this study, we developed a polyurethane/small intestinal submucosa (PU/SIS) hydrogel and investigated its efficacy for accelerating ESD-induced ulcer healing in a canine model. PU/SIS hydrogel possessed great biocompatibility and distinctive pH-sensitive swelling properties and protected GES-1 cells from acid attack through forming a dense film in acidic conditions *in vitro*. Besides, PU/SIS gels present a strong bio-adhesion to gastric tissues under acidic conditions, thus ensuring the retention time of PU/SIS gels *in vivo*. In a canine model, PU/SIS hydrogel was easily delivered via endoscopy and adhered to the ulcer sites. PU/SIS hydrogel accelerated gastric ulcer healing at an early stage with more epithelium regeneration and slight inflammation. Our findings reveal PU/SIS hydrogel is a promising and attractive candidate for ESD-induced ulcer repair.

## Introduction

Stomach cancer is the third leading cause of cancer-related deaths worldwide, with particularly high frequency in Asia [1]. For early gastrointestinal neoplasms, endoscopic submucosal dissection (ESD) is recognized as a preferred treatment, which could successfully achieve a higher en bloc resection rate. However, compared to other endoscopic surgery, ESD is more invasive and takes longer to heal, with pain, bleeding, perforation and other complications [2]. Currently, there is no specific treatment for post-ESD complications, but rather proton pump inhibitors (PPIs), mucosal protective agents and clips are used. However, even with such preventive methods, delayed ulcer healing of >8 weeks has been reported in 5–20% of patients following ESD and the frequency of bleeding associated with post-ESD has been reported up to 38% [3]. Furthermore, the safety and long-term effects of using PPIs are attracting increasing concerns in recent years [4]. Evidence has suggested that chronic use of PPIs may exacerbate small intestinal injury [5, 6] and increase the risk of gastric cancer in patients [7].

More recently, a novel endoscopic tissue shielding biomaterial received widespread attention for ESD-induced ulcer repair, such as polylactic acid, polyethylene glycol membrane, extracellular matrix, etc. [8]. One promising novel biological material that could be used is small intestinal submucosa (SIS). SIS is a type of acellular matrix that has been widely used for scientific and clinical applications, due to its good biocompatibility, biodegradability and low immunogenicity [9, 10]. SIS consists of collagen, proteoglycan glycosaminoglycan, glycoprotein, and a wide variety of growth factors, including vascular endothelial growth factor (VEGF), transforming growth factor (TGF), basic fibroblast growth factor (bFGF) and epidermal growth factor (EGF) [11]. It has been approved by the FDA for hernia repair, cystoplasties, ureteral reconstructions, stress incontinence, etc. [12, 13]. However, rapid degradation and poor mechanical properties of SIS in gastric juice limit the wide application of SIS to gastric tissue repair [12, 14]. Thus, it is necessary to modify SIS for its application in gastric tissue repair.

Polyurethane (PU) is a commonly used medical polymer material with tunable mechanic properties, degradability and good biocompatibility [15]. It has been widely used for soft tissue repair such as blood vessels, skin, heart valve, etc. [16]. Several investigations have been performed on the potential of PU synthesized with acid chain extenders to form controlled release systems for oral administration. It showed that polyurethanes containing carboxylic acid groups exhibit excellent protection of acid hydrophobic and protein drugs from gastric juice in oral drug delivery systems [17, 18]. However, no works have been performed on whether PU can protect the ulcer base under acidic conditions and promote ESD-induced ulcer healing.

Herein, we prepared PU/SIS hydrogel to investigate the feasibility in ESD-induced ulcer repair. We evaluated its physicochemical properties and cell protection under acidic conditions *in vitro*. The ulcer healing effect of the PU/SIS hydrogel was evaluated in a canine ESD-related ulcer model. Our study demonstrates that PU/SIS hydrogel could protect cells from acidic conditions and promote ulcer healing.

## Materials and methods

### Materials

Fresh porcine small intestine was obtained from a slaughterhouse. Polytetramethylene ether glycol (PTMG, M_n_ = 1000), isophorone diisocyanate (IPDI) and 2,2-bis(hydroxymethyl) butyric acid (DMBA) were obtained from Aladdin Industrial Corporation (Shanghai, China) and dried under vacuum before use. All other chemicals were of reagent grade and used as received.

### Preparation and characterization of PU/SIS hydrogel

SIS and PU emulsion were prepared using a previously developed procedure, which is described in detail in the Supplementary Information section [19]. The prepared PU emulsion (21 wt.%) was added dropwise into the SIS matrix solution with continuous gentle shaking at 4°C. Then, the PU/SIS hydrogel was incubated at 37°C, after which a gel formed. The final concentration of SIS in this system is 3% (w/v). Five formulas were prepared: (i) 9 PU/SIS: 9% PU + 3% SIS; (ii) 6 PU/SIS: 6% PU + 3% SIS; (iii) 3 PU/SIS: 3% PU + 3% SIS; (iv) 1.5 PU/SIS: 1.5% PU + 3% SIS; (v) 1 PU/SIS: 1% PU + 3% SIS.

### Fourier-transform infrared spectroscopy (FTIR)

The PU, SIS and PU/SIS hydrogel were determined by FTIR using a Nicolet 6700 FTIR spectrophotometer (Thermo Electron Scientific Instruments, Madison). Spectra were recorded at room temperature in the range of 400–4000 cm^−1^.

### Zeta potential measurements

The electrophoretic mobility values measured by Laser Doppler Velocimetry (LDV) were converted to zeta potential (ZP) values using the Smoluchowski equation. The zeta potential was determined using zeta potential analyzer (Malvern Nano ZSP, Malvern Panalytical). Samples were placed directly into a clear plastic zeta cell and equilibrated for 2 min at 25°C prior to the measurement. The readings for each sample were performed at least three times and at least three batches of each sample were prepared and analyzed.

### Swelling ratio analysis

The swelling ratio of lyophilized PU/SIS hydrogel was measured gravimetrically. In brief, the samples were incubated in 5 ml of phosphate buffer saline (PBS) solution with different pH values at 37°C for 24 h. The surface of the soaked gel was quickly wiped with filter paper and then the mass of each sample was weighed. The swelling ratio of different gels was calculated using the following equation:

$$s\text{welling}\;\text{ratio}\left(\%\right)\;=\left[\left(w_{t}–w_{0}\right)/w_{0}\right]\times 100$$

where w_t_ and w_0_ are the weights of the wet and dry samples, respectively.

### *In vitro* degradation

To mimic the gastric environment, artificial gastric juice, i.e. simulated gastric fluid (SGF) was used in the degradation test *in vitro*. According to the USP Convention, artificial gastric juice was prepared with 0.32% (w/v) porcine pepsin in 0.2% NaCl at pH = 1.2. Approximately 8 mg of the prepared samples were immersed in 1 ml of artificial gastric juice at 37°C and changed every two days. Samples were rinsed with deionized water, lyophilized and weighted (w_0_) at pre-determined harvest time. The average sample mass after incubation was calculated using the results from three different samples.

$$\mathrm{remaining}\;\mathrm{weight}(\%)=\left[{\left(w_{0}-w_{d}\right)/w}_{0\;}\right]\times 100$$

where w_d_ was the sample weight after degradation, and w_0_ was the initial sample weight.

### Biocompatibility study

#### Cytocompatibility

The cytotoxicity of PU/SIS hydrogel was assessed according to ISO standard 10993:2018. Briefly, PU/SIS hydrogels were immersed in high-glucose dulbecco's modified eagle medium (DMEM) (DMEM/H) containing 10% (v/v) Fetal bovine serum (FBS) and 1% (w/v) penicillin–streptomycin in a 5% CO_2_ incubator set at 37°C for 48 h to obtain the extract solution. And 1 × 10^4^ GES-1 cells and L929 cells (Chinese Academy of Sciences) were seeded in each well of a 96-well plate, respectively. After 24 h, the culture medium was replaced by the extract solution, and changed every two days. The cytotoxicity was assessed using Cell Counting KIT-8 according to manufacturers’ instructions (CCK-8, Dojindo).

#### Hemocompatibility

A hemolysis test was performed according to ISO standard 10993:2018. Briefly, 8 ml of fresh venous blood collected from a healthy adult New Zealand white rabbit was diluted with a total of 10 ml of normal physiological saline containing 0.5 ml of potassium oxalate anticoagulant (20 g/L). And then, the fresh venous blood was washed 3–4 times with normal saline until the supernatant was colorless to obtain RBCs suspension. After that, the PU/SIS hydrogel samples (*n* = 3) were added into diluted RBCs suspension (300 μL) and maintained at 37°C for 60 min. Distilled water and normal physiological saline were served as positive and negative controls under the same conditions, respectively. After incubation, samples were centrifuged and the optical density (OD) of supernatant was measured at a wavelength of 545 nm. The hemolysis ratio (HR) was calculated according to the following equation:

$$\text{HR}\left(\%\right)\;=[({\text{OD}}_{t}-{\text{OD}}_{n})/({\text{OD}}_{p}-{\text{OD}}_{n})]\times 100$$

where OD_t_, OD_n_ and OD_p_ mean optical densities of the samples, negative control, and positive control groups, respectively. Test sample with a percentage of more than 5% was considered as hemolytic.

### Protective effect of the PU/SIS hydrogel

GES-1 was used to characterize the protective effect of PU/SIS hydrogel at pH 2 and 4 [20]. 4 × 10^4^ GES-1 cells were seeded in each well of 72 well plates. After 24 h of culture, the PU/SIS hydrogel was added to the above of the 80–90% confluence cells layer. Then, the culture medium adjusted to pH 2 or 4 was added. After 48 h, the CCK8 assay was used to measure cell viability. SIS hydrogel was treated as control.

### Morphological analysis under acidic condition

**Figure 1.**
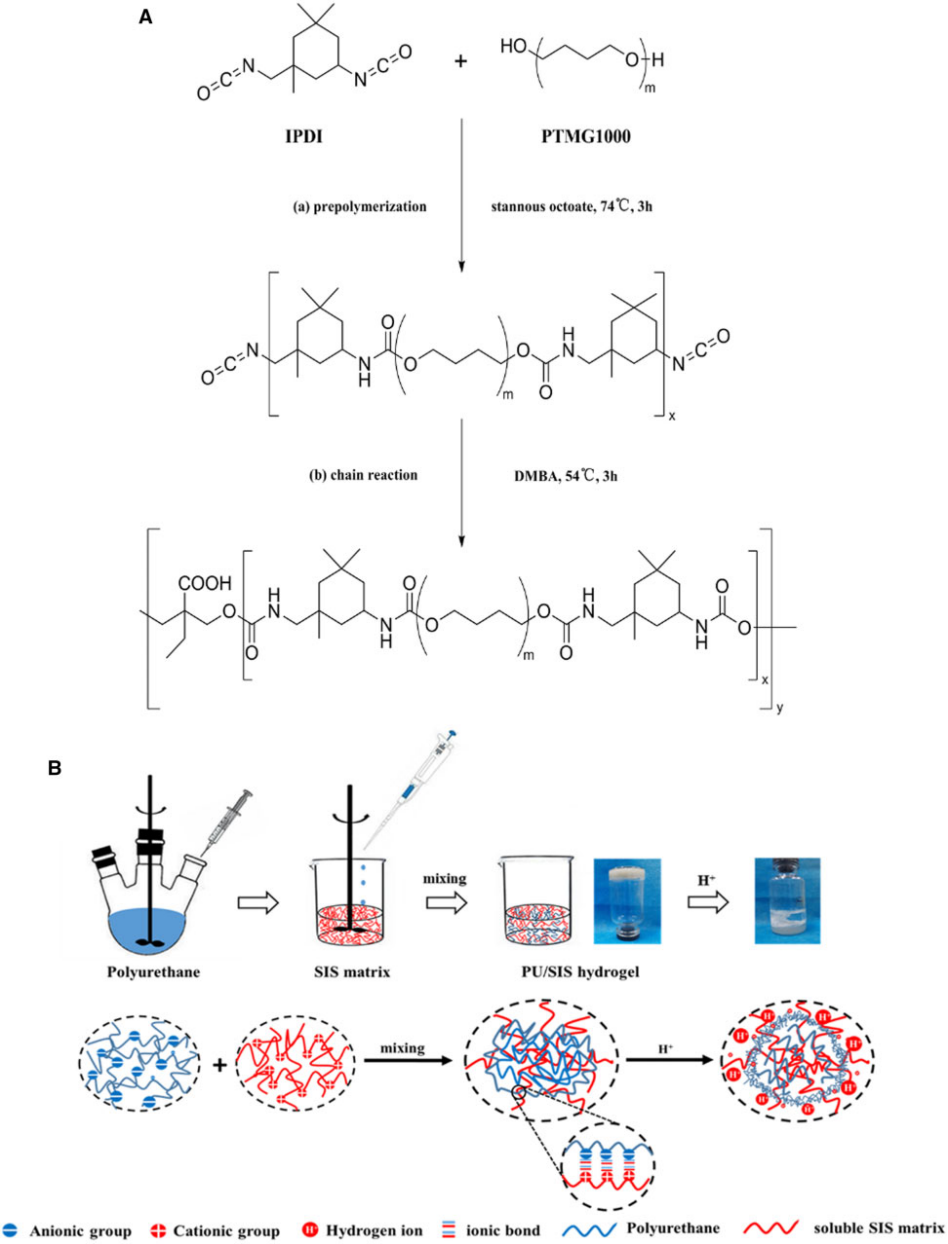
Schematic illustration of the synthesis of PU and PU/SIS hydrogel. (**A**) Molecular structure and synthesis processes of PU. (**B**) The scheme of PU/SIS hydrogel preparation. The prepared PU emulsion (negative charge) was added into soluble SIS matrix (positive charge) dropwise with gentle shaking at 4°C. After well dispersion, the mixture was incubated at 37°C to form PU/SIS hydrogel through ionic bonds. Moreover, a density film was formed on the surface of PU/SIS when hydrochloric acid touches PU/SIS hydrogel.

The surface morphology of PU/SIS hydrogel scaffolds was characterized through scanning electron microscope (SEM) (EVO 10, ZEISS). As shown in Figs. 1 and 4A, the hydrochloric acid solution (pH = 2) was added into PU/SIS hydrogel for 5 min and washed 3 times with PBS. After that, the PU/SIS hydrogel was freeze-dried, cut into 10 mm × 10 mm pieces, coated with Au, and observed under an SEM at a magnification of 30.

### Adhesive ability test

To test the bio-adhesive properties of the PU/SIS hydrogel, lap shear joints between PU/SIS hydrogel and canine gastric tissue blocks (L × W = 5 cm × 1.7 cm) were prepared. Briefly, approximately 0.5 ml hydrogel was applied sparingly to the gap of two pieces of canine gastric tissue which form a junction contact area of 1.7 cm^2^ (l × W = 1.0 cm × 1.7 cm), as shown in Fig. 5A. Subsequently, the lap joint was immersed in simulated gastric fluid (SGF) for 1 min. The end of gastric tissue was clamped to the tensile machine (AG-10TA, Shimadzu) for shear adhesive test and the shear velocity was 50 mm/min [21]. The shear strength was calculated according to the following equation:

$$\mathrm{shear}\;\mathrm{strength}=\;\frac{\mathrm{maximum}\;\mathrm{force}}{\mathrm{junction}\;\mathrm{contact}\;\mathrm{area}}$$

### Animal study

All animal experimental procedures were approved by Sichuan University Animal Care and Use Committee (2015070A), following the Principles of Laboratory Animal Care formulated by the National Society for Medical Research. Twelve dogs were randomly divided into three groups: control group, PPIs group and PU/SIS group.

The model of canine gastric ulcer was established by the ESD technique. A conventional endoscope (GIF-Q260J; Olympus) with a fitted disposable distal attachment cap (D-201; Olympus) was used with a disposable 23G injection needle catheter (Olympus). The animals were fasted for 12 h before the experiment and anesthetized by pentobarbital sodium (1.2 ml/kg). Dots were marked on the posterior wall of the gastric antrum using a dual knife and an electrosurgical unit to identify the margin of the hypothetical lesion, i.e. a circular area 3 cm in diameter. The hydroxyethyl starch was injected into the lateral side of the marker with multi-point injections under mucosa to fully elevate the lesion. A DualKnife (KD-655L, Olympus) was used to cut the mucosa at the distal end of the resected segment to separate the submucosal and intrinsic muscles. Then, ESD-induced ulcer was carefully examined and small vessels were treated with hot hemostatic forceps or electrocoagulation. For PU/SIS group, the PU/SIS hydrogel was initially applied to the site of artificial ulcer with a catheter through the biopsy channel to fully fill the defect. For PPIs group, the animals were treated with proton pump inhibitor drugs (omeprazole 40 mg b.i.d.). For the control group, only ESD was performed and no treatment was applied.

### Endoscopic examination

Follow-up endoscopic examinations were performed at 1, 2, 3 and 4 weeks after the intervention to evaluate ulcer healing and an endoscopic ruler was used to measure the ulcer size. The ulcer index was calculated according to the following equation:

$$\text{ulcer}\;\text{index}=\frac{S_{d}}{S_{0}}\times 100\%$$

where S_d_ was the ulcer size in different time points, and S_0_ was the initial ulcer size.

### Histological analysis

After 2 W and 4 W of follow-up, animals were euthanized by an overdose of IV pentobarbitone according to the surgical facility standard protocols. Gastric specimens were retrieved from both the marginal and central zones. Briefly, the specimens were rinsed with 0.9% NaCl to remove chyme, then fixed overnight with 10% formalin, dehydrated in the graded alcohols and embedded in paraffin. Sections were cut at 3–5 μm and stained with hematoxylin and eosin (H&E). The repair of gastric mucosa was observed by H&E staining. Histological features of inflammation and mucosal morphology were graded according to reported standards (Supplementary Tables S2 and S3) [22, 23].

### Statistical analysis

All the data are presented as the mean ± standard deviation. Statistical analysis was carried out using the Student’s t-test or one-way analysis of variance using SPSS 11.0 software. Differences were considered significant at *P *<* *0.05.

## Results

### Synthesis of PU and preparation of PU/SIS hydrogel

The preparation process of PU and PU/SIS gel is presented in Fig. 1. PU/SIS hydrogel was formed at 37°C and easily delivered by minimally invasive surgical techniques.

**Figure 2.**
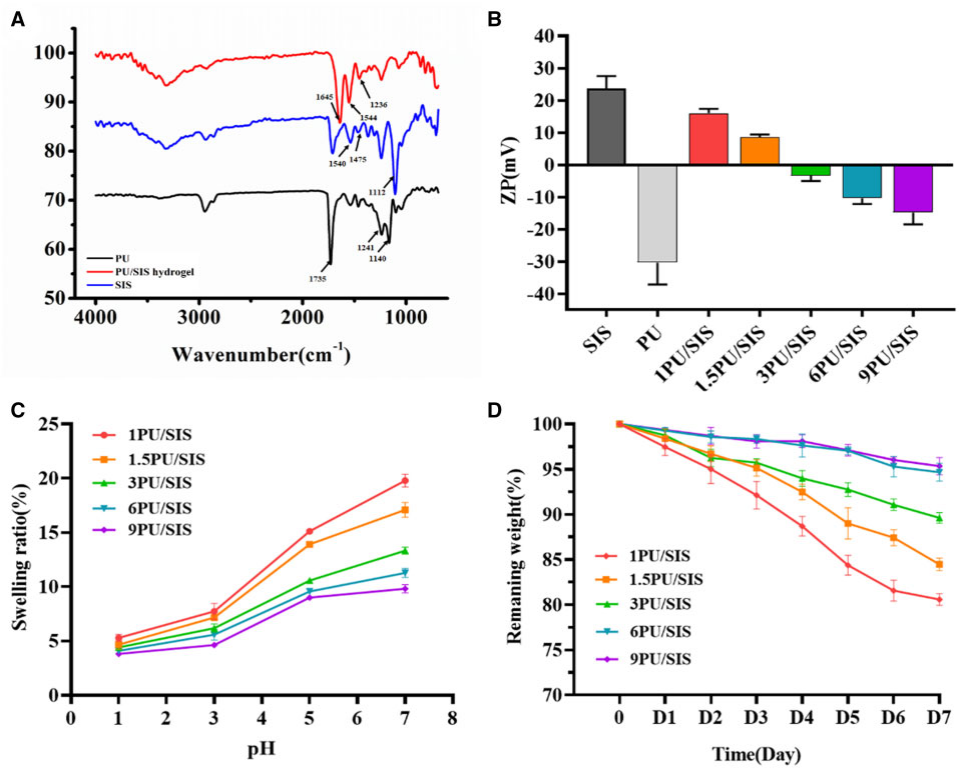
The physicochemical properties of PU/SIS hydrogels. (**A**) Fourier transforms infrared spectra of PU, SIS and PU/SIS hydrogel. (**B**) The zeta potential of PU emulsion, SIS gels and PU/SIS hydrogel. (**C**) The swelling ratios of PU/SIS hydrogel at different pH conditions. (**D**) The degradation properties of PU/SIS hydrogel in SGF (pH = 2).

The FTIR spectra of PU, SIS hydrogel and PU/SIS hydrogel were recorded using Attenuated total reflectance(ATR) mode (Fig. 2A). According to the FTIR spectra recorded for PU, the absorption peak at 1735 cm^−1^, 1241 cm^−1^ and 1140 cm^−1^ was assigned to C = O axial deformations, C–O stretching and N–H stretching, respectively, and the main characteristic bands of isocyanate groups in the range of 2240–2270 cm^−1^ were not detected. All above results confirmed the successful synthesis of PU and the chemical structure was shown in Fig. 1A. On the other hand, the spectrum of SIS showed the presence of collagen containing the main characteristic bands of amide I (1645 cm^−1^), amide II (1544 cm^−1^) and amide III (1236 cm^−1^). Asymmetric stretching of COO^−^ was inconspicuous because of the overlap of the spectra of sodium carboxylate groups (–COONa) with the scissoring of the O–H bonds of absorbed water [24]. In the FTIR spectra of PU/SIS hydrogel, 1540 cm^−1^ and 1475 cm^−1^ assigned to C–O stretching and the characteristic bands of hydroxyl group (920 cm^−1^) were not detected indicating the existence of carboxylate group. Furthermore, the blue shift of the C–N stretching peak (1112 cm^−1^) may be related to the deproteination of carboxyl group binding with ${\text{NH}}_{3}^{+}$ groups of SIS.

### Zeta potential measurements

Zeta potential measurements were performed for PU/SIS mixtures at different mass ratios. Zeta potential analysis displayed that PU emulsion (21 wt.%) had a negative zeta potential value of −30.30 mV and the SIS colloid (3 wt.%) showed a positive zeta potential of +23.60 mV (Fig. 2B). Also, the zeta potential of PU/SIS hydrogel decreased from +20mV to −28.99 mV with the increase of PU contents. The above results indicated the PU and SIS can interact with each other via electrostatic interaction.

### Swelling

The swelling ratios of PU/SIS hydrogel were examined at different pH levels (Fig. 2C). It was seen that the swelling ratios of PU/SIS hydrogel correlated closely with the pH levels. The swelling ratios of PU/SIS hydrogel increased with the increase of pH. These results indicated that PU/SIS hydrogel was sensitive to pH. In addition, it can be observed that a higher SIS content results in higher swelling ratios since SIS is more hydrophilic.

### Degradation

PU/SIS hydrogel was immersed in SGF to determine resistance against degradation *in vitro*. Within the first 12 h, all SIS hydrogels were completely degraded due to H^+^ breaking the network of SIS gel. However, the PU/SIS hydrogel degraded slowly in SGF and the weight loss was not exceeded 20% for 7 days. The weight loss rate gradually decreased as the PU content increased and the remaining weight of 9 PU/SIS and 6 PU/SIS hydrogels were significantly higher than others (Fig. 2D and Supplementary Fig. S1). The present results demonstrated that PU/SIS hydrogel can resist the digestion of SGF and the increasing PU content could reduce the degradation rate of PU/SIS hydrogel.

### The biocompatibility of PU/SIS hydrogel

**Figure 3.**
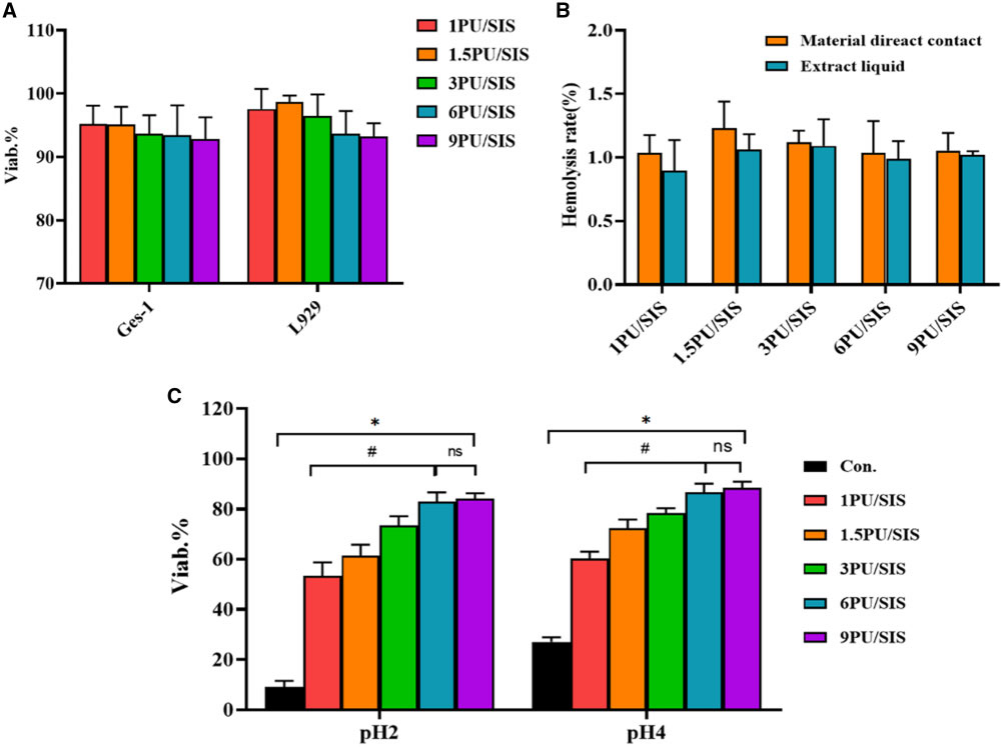
The biocompatibility and protective effect of PU/SIS hydrogel. (**A**) GES-1 cell viability estimated by CCK8 assay after 48 h incubation in different extracts. (**B**) The hemolysis rate (%) evaluated with hydrogels and the corresponding normal saline extract liquids. (**C**) The protective effect of the PU/SIS hydrogels on cell viability *in vitro* under acidic condition. The 6 PU/SIS hydrogel and 9 PU/SIS hydrogel showed better protective effect for GES-1 cells. *: significantly different from control group (*P* < 0.05). #: significantly different from 6 PU/SIS group (*P *<* *0.05).

Cytocompatibility of PU/SIS hydrogel was evaluated by CCK8 assay according to the GB/T 16886.1/ISO 10993-1. As the result showed, the viability of GES-1 and L929 were over 70% with no significant differences between the different groups, indicating that PU/SIS hydrogel extract has no cytotoxicity (Fig. 3A). As indicated by hemocompatibility, PU/SIS hydrogel didn’t lead to erythropoiesis according to the ISO standard (Fig. 3B).

### The effect of PU/SIS hydrogel on cells viability

To evaluate the protective effect of PU/SIS hydrogel on cells in pH 2 and 4 conditions, the viability of GES-1 was tested after 48 h culture. Under the protection of PU/SIS hydrogel, GES-1 showed high viability in acidic conditions (Fig. 3C). In pH 2 conditions, only 11.21% viability was maintained in the control group, but the viability of GES-1 with a statistically significant increase from 53.47 to 84.26% as the PU content increased. However, the difference between 9 PU/SIS hydrogel and 6 PU/SIS hydrogel was not statistically significant. It might indicate that beyond a certain PU content, the protective effect did not increase dramatically and began to stabilize into a plateau region. A similar tendency could be observed in pH 4 conditions. Based on the results, 6 PU/SIS hydrogel was selected for subsequent experiments. Furthermore, an interesting phenomenon was been observed that when the acidic medium was added, the PU/SIS hydrogel formed a layer of tough film on the surface while the underlying hydrogel remained unchanged, which we speculated may be related to the protective effect of PU/SIS hydrogel.

### Morphological analysis in acidic condition

**Figure 4.**
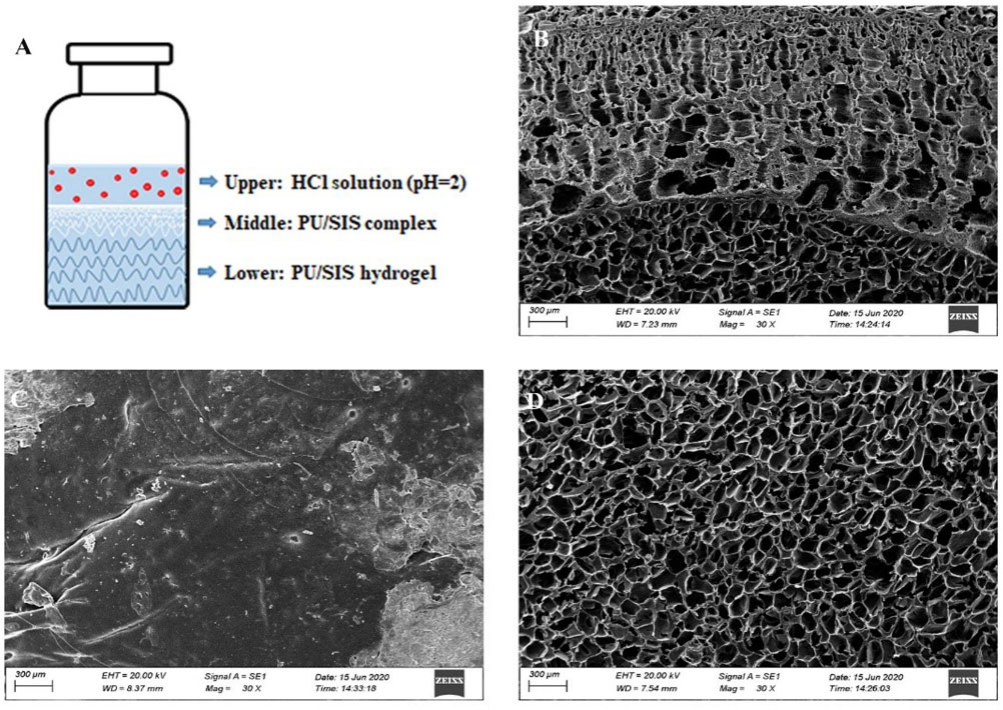
SEM Images of the PU/SIS hydrogel at acidic condition. (**A**) The schematic illustration of SEM sample preparation. (**B**) The top surface of PU/SIS hydrogel exhibited a dense membrane. (**C**) The sectional view of PU/SIS hydrogel showed a complete difference structure between the upper and lower layer. (**D**) The bottom surface of PU/SIS hydrogel had a highly porous structure complex.

To investigate the effect of acid on PU/SIS hydrogel, we evaluated the top surface, the bottom surface and sectional view of PU/SIS hydrogel by SEM. The scheme of the sample preparation was shown in Fig. 4A. The upper layer of PU/SIS hydrogel became a white complex, but the lower layer of hydrogel stayed the same (Fig. 1). As shown in SEM results, the top surface of PU/SIS hydrogel that touches the HCl solution exhibited a compact film (Fig. 4B) and the bottom surface of PU/SIS hydrogel had a highly porous structure (Fig. 4D). Interestingly, it can be observed that the sectional view of PU/SIS hydrogel exhibited a completely different structure between the upper and lower layer (Fig. 4C). The pore size of upper layer of the hydrogel varies regularly. The closer the location to hydrochloric acid, the smaller the pore size. These phenomena indicated that PU/SIS hydrogel effectively prevented H^+^ erosion.

### Bio-adhesive test

**Figure 5.**
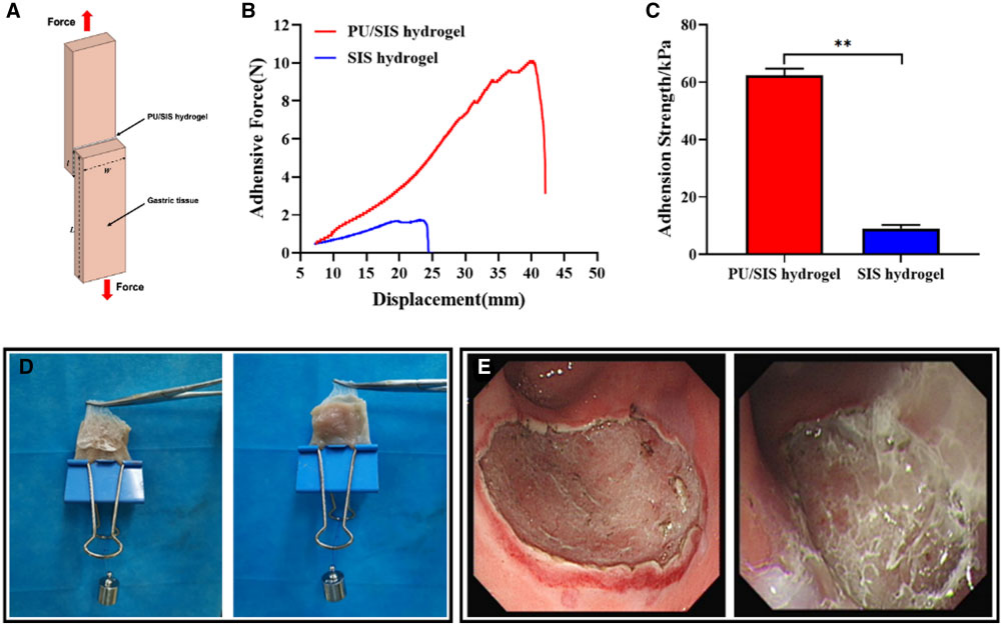
Tissue adhesive properties of PU/SIS hydrogel. (**A**) Schematic illustration of lap shear test. (**B**) Debonding-force-displacement curves of adhesion to hydrogels and gastric tissue. (**C**) The adhesion strength of PU/SIS hydrogel and SIS hydrogel. **: significantly different from control (*P *<* *0.01). (**D**) *In vitro*, PU/SIS hydrogel adhered well on the surface of gastric mucosa. (**E**) *In vivo*, PU/SIS hydrogel could adhere to the ESD surgical site by gastroscopy.

*In vitro*, lap shear measurements were performed to quantify the adhesion of the hydrogels on gastric mucosal surface. Debonding-force-displacement curves for hydrogels and gastric tissues were shown in Fig. 5B. The SIS hydrogels showed negligible adhesion on gastric mucosa, while the PU/SIS hydrogels showed significantly enhanced adhesion strength (Fig. 5C). As shown in Fig. 5D, PU/SIS hydrogel formed a high toughness film that adhered well to the gastric mucosa. *In vivo*, it also has been proved that PU/SIS hydrogel adhered well to ESD-induced ulcers (Fig. 5E). These results suggested that the PU/SIS hydrogel had a strong adhesion to the gastric mucosa, making it potentially useful for gastric ulcer repair application.

### Animal study

**Figure 6.**
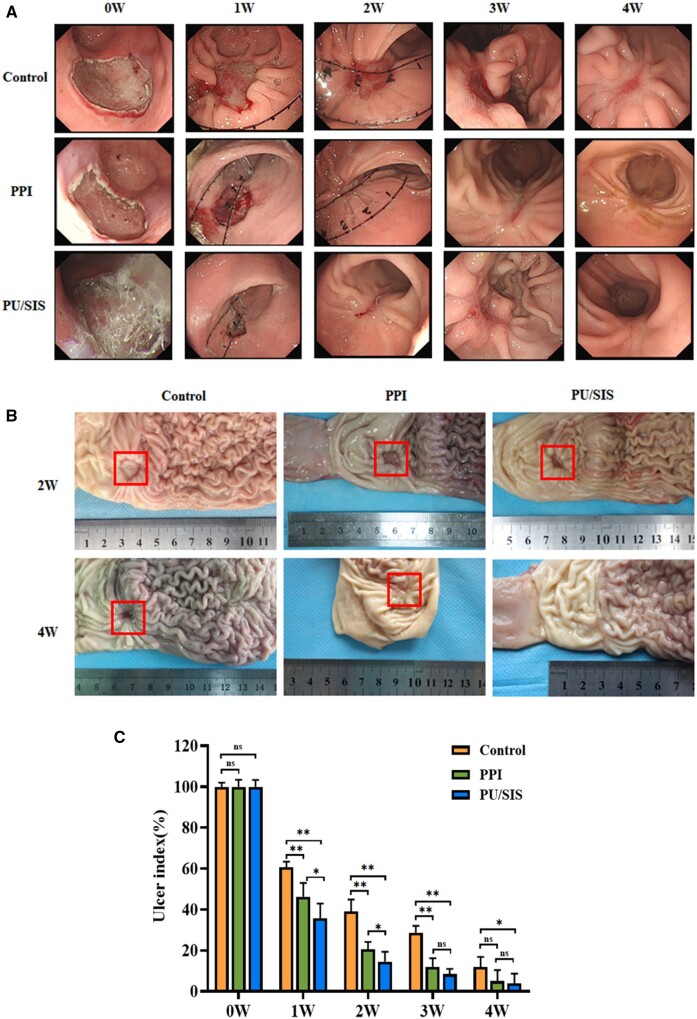
Endoscopic submucosal dissection (ESD)-induced ulcer of the PU/SIS group and the control group. (**A**) Endoscopic surveillance for ulcer healing. (**B**) Gross evaluation of the mucosal surface. (**C**) Quantification of ulcer areas on 1 W, 2 W, 3 W, 4 W post-operation. The PU/SIS hydrogel significantly accelerated ulcer healing at early stage. *: significantly different from control (*P *<* *0.05). **: significantly different from control (*P *<* *0.01).

A total of 12 specimens were safely obtained from beagles. A canine model of artificial gastric ulcer was established by endoscopic submucosal dissection. We recorded the details of ESD procedures that profoundly affected the ulcer healing. There were no significant differences in operative time, cutting speed, and resection area among the groups. Significant bleeding barely occurred, and there was no perforation (Table 1). Endoscopic surveillance and gross examination showed that endoscopically applied PU/SIS hydrogel led to accelerated ulcer healing compared to the controls (Fig. 6). At 2 weeks of surgery, the ulcer size in PPIs and PU/SIS group was less than that in control. At 4 weeks of surgery, the ulcer was completely healed in PU/SIS group while the ulcer was closed until 7 weeks in the control group. The ulcer size at different time points is shown in Supplementary Table S1. It can be seen that the PU/SIS hydrogel prominently accelerated ulcer healing at 1 week after procedure and the ulcer index was significantly lower in PU/SIS group than that in control and PPIs group (Fig. 6C). The ulcer index of the PU/SIS group reduced to 35.82% of the original size at 1 week (versus 60.70% of the control group, *P *<* *0.01; versus 46.24% of the PPIs group, *P *<* *0.05).

**Table 1.** Detail of endoscopic submucosal dissection procedures and statistical analysis

Variable	Control (*n* = 4)	PU/SIS (*n* = 4)	PPIs (*n* = 4)
Surface area of specimen(cm^2^), mean±SD	7.05 ± 0.14	7.41 ± 0.48	7.08 ± 0.23
Procedure time(minutes), mean±SD	22.3 ± 8.31	25.1 ± 10.42	22.1 ± 6.58
En bloc resection, *n* (%)	4 (100)	4 (100)	4 (100)
Major bleeding rate, *n* (%)	0 (0)	0 (0)	0 (0)
Perforation rate, *n* (%)	0 (0)	0 (0)	0 (0)

SD, Standard deviation.

### Histological examination

**Figure 7.**
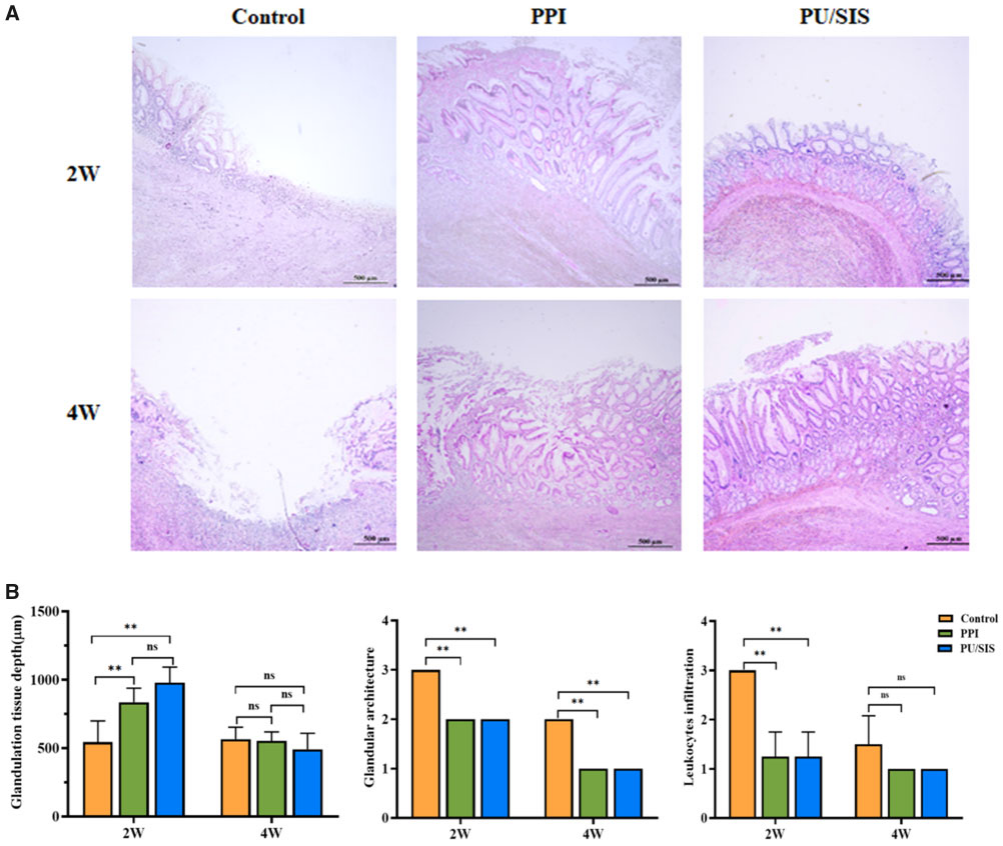
Histological and morphological evaluation of gastric ulcer margin and ulcer base. (**A**) H&E staining of gastric ulcer on 2 W and 4 W post operation, respectively. Scale bars, 500 μm. (**B**) H&E staining and scoring in terms of the ulcer margin granulation tissue depth, and inflammatory infiltration. *: significantly different from control (*P *<* *0.05). **: significantly different from control (*P *<* *0.01).

Histology was evaluated in terms of inflammation and mucosal regeneration. At 2 weeks after surgery, epithelial cells migrated to the ulcer margin forming a ‘healing zone’ in all groups, and the base of ulcer was covered by granulation tissue (Fig. 7A). The granulation tissue depth was significantly higher in PPIs and PU/SIS group than that in control group (Fig. 7B, *P *<* *0.01). There was very little epithelium regeneration in the ulcer base of the control group while considerably more regenerative epithelium was observed in PPIs and PU/SIS group. Meanwhile, the grade of inflammatory infiltration was reduced in PU/SIS and PPIs group compared to the control group at 2 weeks (Fig. 7B and Supplementary Fig. S2, *P *<* *0.01). At 4 weeks, more regenerative epithelium with normal morphology and glands was observed around the ulcer base in PPIs and PU/SIS group, whereas epithelial cells were poorly differentiated in the control group. The scores of the glandular architecture were significantly different between the PU/SIS hydrogel group and the control group at 4 weeks (Fig. 7B and Supplementary Fig. S3, *P *<* *0.05).

## Discussion

Intragastric pH profoundly affects the progress of gastric ulcer healing. Increasing the intragastric pH to protect ulcer lesion from gastric juice erosion is the key to accelerating ulcer healing and reducing the frequency of post-operative bleeding and perforation [25]. Herein, we constructed PU/SIS hydrogel to explore the therapeutic potential for ESD-related ulcers in a canine model. We found that PU/SIS hydrogel could protect GES-1 cells at acidic conditions, adhere to gastric mucosa and accelerate ulcer healing in the ESD-related ulcer canine model. These results indicated that the PU/SIS hydrogel could be a promising biomaterial for the endoscopic treatment of ESD-induced ulcers.

SIS consists of predominantly collagens plus glycosaminoglycans, proteoglycans, fibronectin and active factors [11]. It has been reported that SIS hydrogel could release growth factors sustainably and promote angiogenesis *in vitro* and *in vivo* [26]. It has been widely applied to the repair of various tissues and achieved great effects. However, the rapid degradation of SIS hydrogel in gastric juice limits their use in gastric ulcers, and no work has been published to date on SIS hydrogel for gastric ulcer repair. Herein, we compounded SIS hydrogel and PU to construct PU/SIS hydrogel. We found that the addition of PU did not affect the gel formation. According to the zeta potential results, the SIS soluble matrix carried positive charge while the PU emulsion carried negative charge and the zeta potential of PU/SIS complex was decreased as the PU content increased. These results indicated that SIS and PU have a tendency to form electrolyte hydrogel through ionic bonds. Moreover, the FTIR spectra of PU/SIS hydrogel also proved the existence of carboxylate group that complexed with ${\text{NH}}_{3}^{+}$ through noncovalent bonding. The procedure and principle for constructing the PU/SIS hydrogel were shown in Fig. 1B.

The biomaterial for treating ESD-induced ulcer is often required to obtain properties including good biocompatibility, ease of delivery, suitable remaining time on the ulcer, good adhesion to ulcer region, promoting wound healing, etc. [20]. Many previous studies have shown that protecting gastrointestinal ulcers from gastric juice could effectively promote ulcer healing [20, 27]. It is most closely related to gastric ulcer healing rates that suppress the erosion of gastric juice at 24 h postoperation [25]. Our results proved that PU/SIS hydrogel could significantly improve the viability of GES-1 cells under low pH culture conditions compared to pure SIS gel. The trend of significantly higher cell protection ability with higher PU content of PU/SIS hydrogel was observed (Fig. 2E). When the PU content reaches 6%, PU/SIS gel effectively protects cells from acid attack. These results indicate that PU plays an important role in protecting cells from acidic conditions. We speculated that it may relate to the PU/SIS film formation in the acidic condition. When the acidic medium was added to PU/SIS hydrogel containing cells, the gel surface rapidly coagulated into a dense film, while the underlying gel was unaffected by the acidic conditions (Fig. 1B). In previous studies, polymers containing carboxyl group has been proved to be sensitive to pH [28]. Many examples of PU used for the delivery of proteins and oral drugs have been reported [17, 18]. The carboxylic acid groups of PU were protonated in acidic conditions, and the PU/SIS coacervate rapidly formed a dense membrane (Fig. 1B). The dense membrane could separate the cells from the acidic environment avoiding acid attack. To prove the structure change of PU/SIS hydrogel under acidic conditions, we scanned the microscopic morphology of PU/SIS gels by SEM. According to the SEM results, the PU/SIS gels form a film on the surface and the closer to the surface the gels coacervate to form smaller pores, while the underlying gels do not differ in their microstructure from those under neutral conditions. In addition, a similar phenomenon was observed in the degradation tests where the surface of PU/SIS hydrogel was covered with white PU/SIS film in acidic conditions. The degradation results displayed that higher the PU content, lower the degradation rate (Fig. 2D), while the 6 PU/SIS hydrogel showed no significant difference from 9 PU/SIS hydrogel. We believe that the change in degradation rate is also related to the change in PU/SIS structure under acidic conditions. Since the membrane formed on the gel surface is mainly caused by PU protonation, when the PU content is increased, the PU/SIS membrane formed on the surface was denser and could reduce the degradation rate of SGF more effectively. These above results indicated that PU/SIS gels are effective in blocking the erosion of gastric juice under acidic conditions.

Great bio-adhesion is essential to biomaterial for gastrointestinal ulcer repair since the shear stress exerted by the movement of gastric contents affects the persistence of biomaterial. At present, most biomaterials used for ulcer treatment are usually fixed by Endoclips, but few works consider the adhesive properties. In this study, we measured the adhesive strength through the lap shear measurements. Our results suggested that PU/SIS hydrogel could adhere well to gastric mucosa and the adhesion strength of PU/SIS hydrogel was significantly higher than SIS hydrogel (Fig. 5). The surface of biological tissue is more complex, containing positive and negative charges, polar and non-polar groups [29]. The formation of bio-adhesion is usually based on intramolecular hydrogen bonding and ionic bonds, but it is hard to establish hydrogen or electrostatic bonding with natural tissue in wet environment [30]. Thus, conventional hydrogel has poor adhesion to biological surfaces [21]. Previous reports have proved that PU is a good adhesive since it contains high polar group content, such as urethane and urea [31]. These polar groups could offer stronger hydrogen bonds, leading to a stronger adhesive strength. On the other hand, biological tissues usually have negatively charged surfaces [21], whereas PU/SIS are protonated under acidic conditions and positively charged surfaces. Therefore, strong adhesion based on electrostatic interactions between natural tissues and PU/SIS hydrogels is prone to form under acidic conditions. Besides, the hydrogen bonds and electrostatic interactions could not cause any tissue damage. Gastric peristalsis exerts tension on PU/SIS hydrogel attached to stomach wall during digestion, which influences the application for gastric ulcers. Regrettably, we have not yet obtained the mechanical properties of PU/SIS hydrogel, because the coacervation of PU/SIS hydrogel was too fast to form regular films, which couldn’t produce a standard mechanical specimen.

According to the report, the phase of gastric ulcer healing is mainly divided into three phases which consist of slow healing in the first so-called lag phase (stage A), rapid healing in the second phase (stage B), and, again, slow healing in the third so-called remodeling phase (stage H) [32]. Howden and Hunt demonstrated the relationship between the suppression of acidity and their corresponding ulcer-healing rates [33]. They found that reducing intragastric acidity in the early stage could significantly accelerate ulcer healing rates. Another work by Xia *et al*. also demonstrated that the early promotion of ulcer healing at day 7 might have greater clinical relevance [22]. In the current study, we observed enhanced ESD-induced ulcer healing and a reduction in mean ulcer size to 35.82% of the original size in PU/SIS group within 1 week post-operatively, versus 46.24% of the PPIs group (*P *<* *0.05). Furthermore, the grade of inflammatory infiltration was significantly lower in PU/SIS group and PPIs group than that in control at 2 weeks after operation. These results indicated that PU/SIS hydrogel could accelerate the gastric ulcer repair with increasing healing rate and reducing inflammatory infiltration in the early stage. In addition, a small amount of PU/SIS hydrogel was found to adhere to the ulcer surface only at 1 week after surgery. This might be due to epithelium regeneration which breaks the hydrogen bonds between PU/SIS hydrogel and the gastric mucosa. Based on these phenomena, we speculated that PU/SIS hydrogel played a major role in the early stages of ulcer healing.

In summary, PU/SIS hydrogel successfully resisted gastric juice erosion and provided a friendly microenvironment for ulcer healing. Furthermore, PU/SIS hydrogel could be implanted through gastroscopy to achieve the integration of diagnosis, treatment and repair. Additionally, the protective effect of PU/SIS hydrogel may play an important role in mucosa repair, although a clear mechanism needs to be studied further.

## Conclusion

In this study, we have demonstrated the feasibility of PU/SIS hydrogel in ESD-induced ulcer repair. PU/SIS hydrogel possessed pH-sensitive property, good biocompatibility, better bio-adhesion and protective effect for gastric epithelial cells. It has been proved that PU/SIS hydrogel plays an important role in ulcer repair at early stage with slight inflammatory infiltration and lower ulcer index. Based on these results, PU/SIS hydrogel can be a promising biomaterial for the endoscopic treatment of ESD-induced ulcer.

## Supplementary data

Supplementary data are available at *REGBIO* online.

*Conflict of interest statement.* None declared. 

## Funding

This work was supported by The National Key R&D Program of China (2017YFC1104702), Sichuan Science and Technology Program (2019JDRC0020), and the 1.3.5 Project for Disciplines of Excellence, West China Hospital, Sichuan University (ZYJC18002). 

## Supplementary Material

rbaa056_Supplementary_Data
