# Supplementary material for: Accelerating ESD-induced gastric ulcer healing using a pH-responsive polyurethane/small intestinal submucosa hydrogel delivered by endoscopic catheter
Source: Regen Biomater. 2021 Jan 4;8(1):rbaa056. doi: 10.1093/rb/rbaa056 (PMC7947578; doi:10.1093/rb/rbaa056)
Supplement: rbaa056_Supplementary_Data [file rbaa056_supplementary_data.docx]

**Supplement Information**

**Preparation of PU/SIS hydrogel**

SIS powder and PU emulsion were prepared using a previously developed procedure. PU prepolymer was synthesized through a bulk polymerization procedure. IPDI and PTMG were polymerized to form pre-polymers under the catalysis of stannous octoate. Subsequently, the products of pre-polymer reacted with chain extender (DMBA) were neutralized with trimethylamine (TEA) and emulsified with high speed stirring. The final concentration of PU was 21 wt.%. The lyophilized SIS was crushed in liquid nitrogen using a freezer mill (6700, SPEX; Metuchen, NJ) into 20μm-sized powder. The 3 w/v % SIS powder was digested in an aqueous solution (3% (v/v) acetic acid and 0.1% (w/v) pepsin) for 48 h to obtain homogeneous SIS solution. Then, the homogeneous SIS solution was freeze-dried again at -70℃, cut into flocculent pieces and sterilized using ethylene oxide (EO) gas to obtain soluble SIS matrix. The soluble SIS matrix was dissolved in PBS that containing 0.1M NaOH at 4℃. Then, PU emulsion (21 wt.%) was added dropwise into the SIS matrix solution with continuous gentle shaking at 4℃. Finally, the PU/SIS hydrogel was incubated at 37 °C, after which a gel formed. The final concentration of SIS in this system is 3% (w/v). Five formulas were prepared: 1) 9 PU/SIS: 9% PU + 3% SIS; 2) 6 PU/SIS: 6% PU + 3% SIS; 3) 3 PU/SIS: 3% PU + 3% SIS; 4) 1.5 PU/SIS: 1.5% PU + 3% SIS; 5) 1 PU/SIS: 1% PU + 3% SIS.


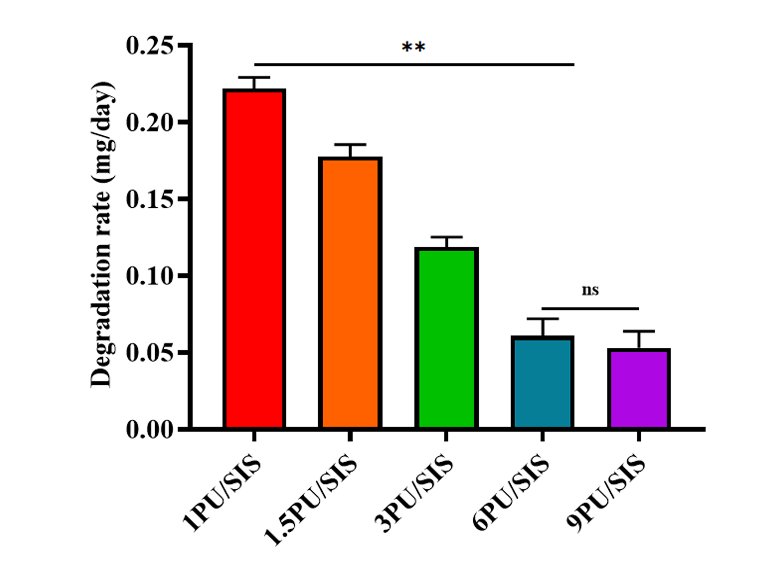


**Figure S1**. Average degradation rate of PU/SIS hydrogel in SGF. As the PU content increased, the degradation rate of PU/SIS hydrogels decreased, while there was no significant difference between the degradation rates of 6 PU/SIS and 9 PU/SIS.


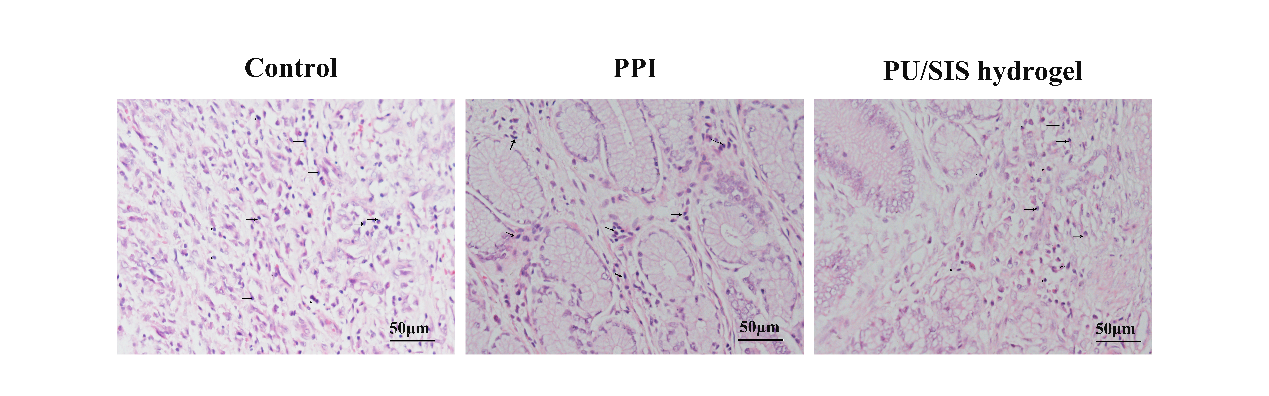


**Figure S2**. Inflammatory infiltrates of the ulcer base at 2 weeks postoperatively. Black arrow indicated inflammatory cells. Scale bars, 50μm.


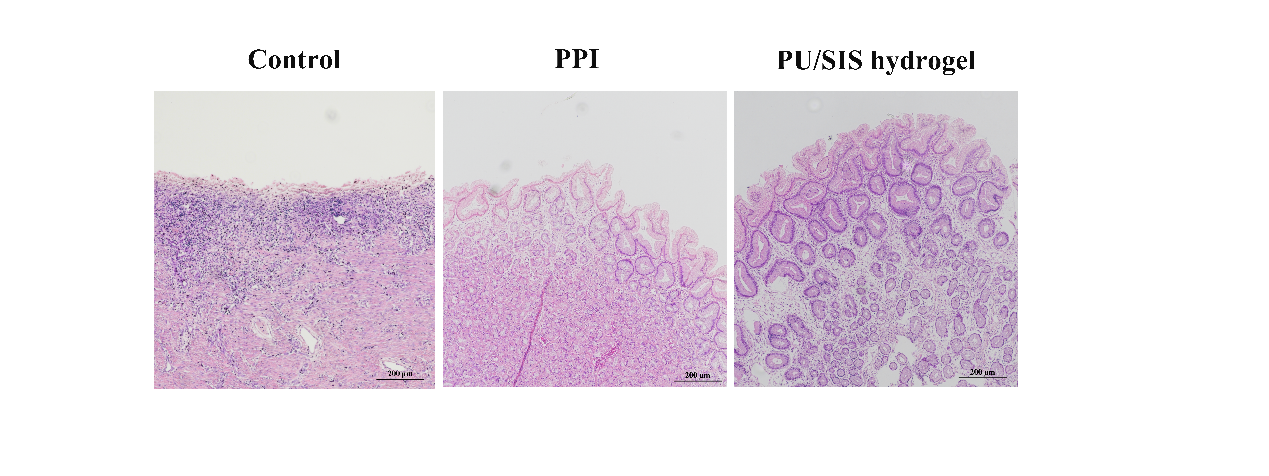


**Figure S3**. Glandular structure of the ulcer base at 2 weeks postoperatively. Scale bars, 200 μm.

Table S1. Qualification of ulcer size (cm^2^) at different time points

|  | Control (n=4) | PPIs (n=4) | PU/SIS (n=4) |
| --- | --- | --- | --- |
| 0W | 7.05±0.14 | 7.08±0.23 | 7.12±0.23 |
| 1W | 4.28±0.19 | 3.28±0.47 | 2.55±0.50 |
| 2W | 2.75±0.41 | 1.46±0.25 | 1.03±0.35 |
| 3W | 2.01±0.24 | 0.85±0.30 | 0.60±0.19 |
| 4W | 0.84±0.35 | 0.35±0.38 | 0.34±0.34 |

All data are shown as the means ± SD.

Table S2. Grade of inflammatory infiltrates

| Grade | Inflammatory infiltration |
| --- | --- |
| 0 | No inflammatory cells |
| 1 | < 10 inflammatory cells /HPF |
| 2 | > 10 inflammatory cells /HPF with infiltration limited to ≤50% of mucosal surface |
| 3 | Infiltration involving > 50% of mucosal surface |

Table S3. Grade of mucosa morphology

| Grade | Mucosal morphology |
| --- | --- |
| 0 | Normal glandular structure |
| 1 | Gastric glands arrange sparsely with some slightly dilated |
| 2 | Morphologic changes between grades 1 and 3 |
| 3 | Gastric glands are dilated markedly with distorted architecture and epithelial cells are poorly differentiated |
